# Effects of Turmeric Powder on Aflatoxin M1 and Aflatoxicol Excretion in Milk from Dairy Cows Exposed to Aflatoxin B1 at the EU Maximum Tolerable Levels

**DOI:** 10.3390/toxins14070430

**Published:** 2022-06-24

**Authors:** Flavia Girolami, Andrea Barbarossa, Paola Badino, Shiva Ghadiri, Damiano Cavallini, Anna Zaghini, Carlo Nebbia

**Affiliations:** 1 Department of Veterinary Sciences, University of Turin, Largo Braccini 2, 10095 Grugliasco, Italy; paola.badino@unito.it (P.B.); shiva.ghadiri@yahoo.com (S.G.); carlo.nebbia@unito.it (C.N.); 2 Department of Veterinary Medical Sciences, University of Bologna, Via Tolara di Sopra 50, 40064 Ozzano dell'Emilia, Italy; andrea.barbarossa@unibo.it (A.B.); damiano.cavallini@unibo.it (D.C.); anna.zaghini@unibo.it (A.Z.)

**Keywords:** turmeric powder, curcumin, curcuminoids, aflatoxin B1, aflatoxin M1, aflatoxicol, milk, dairy cows

## Abstract

Due to the climatic change, an increase in aflatoxin B1 (AFB1) maize contamination has been reported in Europe. As an alternative to mineral binders, natural phytogenic compounds are increasingly used to counteract the negative effects of AFB1 in farm animals. In cows, even low dietary AFB1 concentrations may result in the milk excretion of the genotoxic carcinogen metabolite aflatoxin M1 (AFM1). In this study, we tested the ability of dietary turmeric powder (TP), an extract from Curcuma longa (CL) rich in curcumin and curcuminoids, in reducing AFM1 mammary excretion in Holstein–Friesian cows. Both active principles are reported to inhibit AFM1 hepatic synthesis and interact with drug transporters involved in AFB1 absorption and excretion. A crossover design was applied to two groups of cows (*n* = 4 each) with a 4-day washout. Animals received a diet contaminated with low AFB1 levels (5 ± 1 µg/kg) for 10 days ± TP supplementation (20 g/head/day). TP treatment had no impact on milk yield, milk composition or somatic cell count. Despite a tendency toward a lower average AFM1 milk content in the last four days of the treatment (below EU limits), no statistically significant differences with the AFB1 group occurred. Since the bioavailability of TP active principles may be a major issue, further investigations with different CL preparations are warranted.

## 1. Introduction

Aflatoxins (AFs) are widespread dangerous mycotoxins synthesized by *Aspergillus flavus* and *A. parasiticus* that contaminate several food and feed commodities [1]. While the optimal conditions for toxinogenesis occur in tropical and subtropical regions, in the last decades, AFs have become a concern in southern EU countries. Among the several factors responsible for the significant increase of maize contamination, a number of studies report the major role played by climate change [2,3]. AFs display a large array of toxic effects upon the bioactivation by drug-metabolizing enzymes, particularly cytochrome P450 (CYP)3A and 1A, which are involved in the generation of both AFM1 and the most toxic AFB epoxide (AFBO), a reactive genotoxic hepatocarcinogen responsible also for cytotoxic effects and oxidative stress [4,5]. In its latest evaluation, IARC classified AFs (AFB1, AFB2, AFG1, AFG2A and AFM1) as carcinogenic to humans [6].

Among AFs, AFB1 is the most important compound in terms of prevalence and toxicity for both humans and farm animals; in addition, AFM1 is the main toxic metabolite of AFB1, found in milk and dairy products from AF-exposed dairy cows, posing a further health risk for the consumer [7]. An additional metabolic pathway entails the NADPH-dependent generation of aflatoxicol (AFL), a reduced metabolite which is considered less toxic than AFB1 but may be easily re-converted to the parent compound by liver dehydrogenases, thus prolonging the persistence of the mycotoxin in the body [8]. Variable amounts of AFL may be also found in milk and cheese [9]. Interestingly, in vivo and in vitro studies indicate that efflux transporters like ABCG2 (also referred to as BCRP1) are involved both in the enteric absorption of AFB1 and in the mammary excretion of AFB1 and its metabolites [10,11].

To protect animal and human health, a maximum residue limit of 20 μg AFB1/kg in feed materials and of 5 μg AFB1/kg in compound feed for dairy cattle have been set in the EU (EU Regulation 574/2011), along with a limit of 0.05 μg AFM1/kg for dairy milk (EU Regulation 165/2010). A widely applied method to reduce AFB1 bioavailability in contaminated feed is the use of binders (e.g., clays, aluminosilicates, yeast cell walls and many others) able to form complexes with the toxins, thereby mainly decreasing their enteric absorption [12]. However, the use of binders is not free from potential side effects due to their capacity to adsorb amino acids, vitamins and possibly other nutrients or feed additives [13]. There is, therefore, an increasing interest toward natural compounds with the potential to mitigate AFB1-mediated adverse effects through different mechanisms, reducing the accumulation of its metabolites in the tissues of different food-producing species as well as AFM1 milk contamination in ruminants [12,14,15,16,17]. Among phytogenic products, curcumin (CUR) and curcuminoids (CURS, i.e., desmethoxycurcumin and bis-desmethoxycurcumin) from turmeric (*Curcuma longa*) are receiving growing attention, based on their numerous beneficial properties including anti-inflammatory, antineoplastic and antioxidant effects in humans and in various animal species [18,19]. Such properties, particularly the ability displayed by turmeric derivatives of acting as free radical scavengers, are well recognized [7]. Despite that, turmeric extract (containing at least 90% active substances as the sum of CUR and CURS) has been authorized in all animal species in the EU for use as a sensory (i.e., flavoring) agent; in dairy cows, a maximum content of 15 mg turmeric extract/kg complete feed is recommended (EU Regulation 2021/551).

Interestingly, CUR has the potential to both lower the generation and enhance the in-activation of toxic AF metabolites through the modulation of phase I and phase II bio-transformation enzymes participating in AF metabolism [20,21]. CUR has also been reported to interact with drug transporters like ABCB1, ABCC1 and especially ABCG2 in human cell lines [22], as well as with ABCB1 in chick intestine [23], thus potentially affecting absorption and/or mammary excretion of AFB1 and its metabolites (see above).

The in vivo CUR- and CURS-mediated amelioration of AFB1 toxicity has been documented in rats and broiler chicks (for a review see [21]), but little is known on the ability of either compound in reducing AFM1 synthesis and/or milk excretion in the bovine species. In a fetal bovine hepatocyte cell line, CUR and CURS were recently reported to significantly depress both the AFB1-mediated cytotoxicity and AFM1 synthesis along with a decrease in CYP3A28 activity [24]. 

The main purpose of this study was to assess whether, based on the reported effects on AFB1 metabolism, absorption and excretion, CUR and CURS from turmeric powder (TP) were able to decrease AFM1 and AFL milk content in lactating dairy cows exposed to dietary AFB1 concentrations approaching the maximum limits set by the EU (5 μg kg/compound feed). Effects on milk yield and milk characteristics were also investigated.

## 2. Results and Discussion

### 2.1. Effects on Milk Yield, Milk Composition and Somatic Cell Count

No statistically significant treatment-related differences in all the examined milk parameters were found at any time points (Table 1 and Table S1). 

Under the adopted experimental conditions (5 ± 1 µg AFB1/kg for 10 days corresponding to approximately 0.13 µg AFB1/kg bw/day), milk yield, milk composition and somatic cell count (SCC) were not affected by the AFB1-contaminated diet. This was not unexpected since, in other experiments, all the above milk parameters remained unaltered by the exposure of dairy cows to even higher dietary AFB1 levels and, in certain instances, for a longer period of time. For example, this was the case for animals offered a diet containing 20 or 40 µg AFB1/kg for 7 weeks [25,26] or 100 µg AFB1/kg (DM) for 14 days [27]. Likewise, no changes in milk yield and SCC (the only tested milk parameters) were reported in dairy cows’ dietary exposed to 0.16 µg AFB1/kg bw/day for 10 days [28], i.e., an experimental protocol very similar to that adopted in the present study. A significant decrease in milk yield and increase in SCC should be expected only at very high daily AFB1 exposure (>~20 µg/kg bw) [29]. Little is known about the effects of phytonutrients in dairy ruminants (reviewed by [30]), and scant data are available concerning the effects of CUR and CURS on milk parameters in dairy cows. There was a slight but statistically significant decrease in milk yield and in SCC (N.S.) in Holstein cows intra-abomasally administered with 2000 mg turmeric oleoresin (1900 mg CURC)/head for 9 days [31].

### 2.2. Effects on Milk Concentration of AFM1

**Figure 1:**
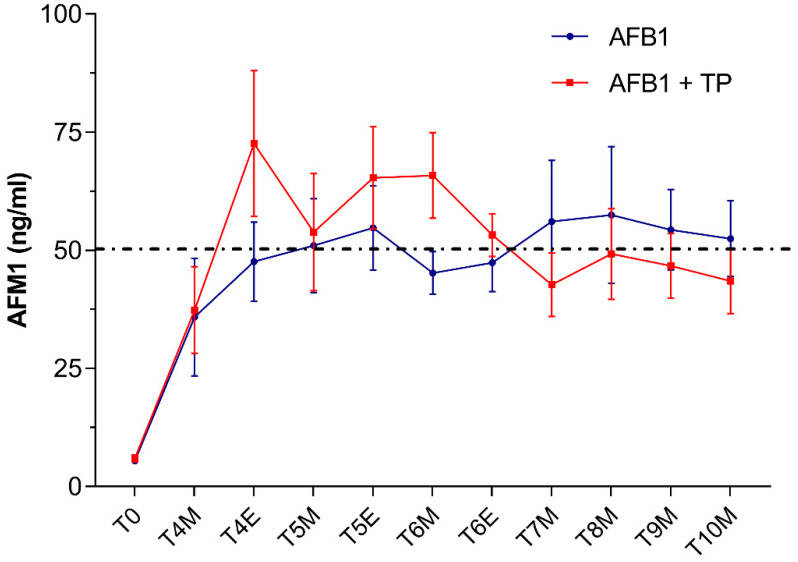
Time course of AFM1 excretion in milk from cows administered AFB1 or AFB1 + TP. Data are expressed as mean ± SEM (*n* = 8). M = morning; E = evening.

Very low AFM1 levels averaging 8 ± 3 μg/L were detected in milk samples collected in cows from both groups at T0 (i.e., 3 days before starting AFB1 administration). By contrast, as early as 24 h after feeding the AFB1-contaminated diet (T4), AFM1 levels raised up to mean concentrations around 36–37 ng/L (Figure 1). 

These results are consistent with a rapid absorption and biotransformation of the mycotoxin followed by the prompt milk excretion of AFM1, as documented in previous studies performed in dairy cows [28,32]. In our study, in both AFB1 and TP animals, the exposure to AFB1 concentrations around the upper limit of EU legislation in compound feed for dairy cattle (5 μg/kg) resulted in AFM1 average milk levels which, at some time points, slightly exceeded the maximum tolerance of 0.05 μg/L established by the EU. Based on recent surveys in EU countries [7,33], the likelihood of bulk milk AFM1 contamination at levels higher than the statutory limits should be considered very low under field conditions. However, as was the case in our study, violative residues of AFM1 (>0.05 μg/L) may be detected under certain circumstances in milk samples from dairy cows fed with rations approaching the EU limits of 5 μg/kg [34,35]. It is worth noting that the amount of AFM1 excreted into milk is affected by several factors including, among others, milk yield, stage of lactation and individual variation [28] (and many references therein). 

In our study, no statistically significant differences in AFM1 milk concentrations occurred between cows supplemented with TP (group AFB1 + TP) and cows receiving AFB1 alone (group AFB1). Of note, in the second part of the study (from T6 to T10), average AFM1 milk values (ng/L) were slightly lower in cows supplemented with TP (46 ± 6) than in cows receiving AFB1 alone (53 ± 8), being thus, on average, below the maximum EU permitted levels of 0.05 µg/L (Figure 1). In the first part of the trial and until T6, TP cows had instead average AFM1 milk levels slightly higher than AFB1 cows. This seeming dual effect of TP on milk AFM1, if confirmed by future experiments, might reflect CUR and CURS interactions with drug transporters [22,23] or with CYPs involved in AFB1 biotransformation. Previous in vitro investigations using a bovine hepatocyte cell line reported that either CUR or CURS (at 10 μM) proved effective in decreasing CYP3A28 expression and activity [36] and AFM1 synthesis up to more than 50% [24]; a similar trend was observed in a bovine mammary epithelium cell line [37]. 

The reasons underlying the in vivo negligible efficacy of active principles from TP in reducing the AFM1 content in dairy milk from AFB1-exposed cows are not known, and to the best of our knowledge, no similar studies are available in the open literature. Bioavailability of CUR and CURS may be an issue. While there is data on phytochemicals such as flavonoids [38] or other polyphenols [39], no specific studies on the kinetics of turmeric derivatives have been published in ruminant species [7,40]. The only available information is limited to laboratory species and humans [41]. The kinetics of CUR in animal models and humans has been recently reviewed [7,42]. In short, both CUR and CURS undergo an extensive and complex degradation at enteric and hepatic levels, mainly involving the generation of several reduced metabolites; both the parent compounds and the generated metabolites are then subjected to sulphonation and glucuronidation and excreted mostly in the feces and, to a very small extent (1–2%), in the urine. The extensive first pass metabolism entails a very low oral bioavailability, which has been estimated around 1%. 

In our study, the administration of 20 g TP (approximately corresponding to an intake of 500 mg CUR and CURS/head/day for 10 days and 0.66 mg/kg bw/day) did not substantially affect the AFM1 content in dairy milk in cows exposed to low dietary AFB1 concentrations. While no similar studies performed in dairy ruminants have been published so far, it should be noted that CUR-related beneficial effects have been documented in other food-producing species, including ruminants. For example, an improvement in both serum antioxidant status and in the immune response was detected in rams orally receiving 450 mg/head/day CUR for 4 weeks, corresponding to approximately 17 mg curcumin/kg bw/day [43]. Likewise, the inclusion of 150 mg CUR/kg diet for 14 days, corresponding to about 300 mg/kg bw/day, proved effective in preventing liver toxicity and AFBO-DNA adducts formation in AFB1-treated broiler chicks [44]. As mentioned above, an average intake of 0.66 mg CUR and CURS/kg bw/day could be calculated in cows from the present study. Therefore, in addition to possible differences due to the use of the pure substance (CUR) in the cited reports instead of a turmeric derivative, the observed lack of significant treatment effects on AFM1 milk content might be attributable to the much lower dosages used in our experiment. In this respect, however, it should be stressed that the CUR and CURS selected dosages were below the estimated maximum safe feed concentration of approximately 720 mg/day calculated by EFSA [7].

### 2.3. Effects on Milk Concentration of AFL

AFL concentrations in dairy milk are depicted in Table 2. Five out of eight cows exposed to AFB1 only showed the presence of trace concentrations (<12 ng/L) of AFL in milk at one time point at least, but measurable levels (>12 ng/L) were found in only two cows. In cows supplemented with TP, AFL trace concentrations at one time point at least occurred in three individuals only, and AFL levels higher than the LOQ (12 ng/L) were detected in none of them. 

Scant information is available on the formation of AFL in cattle as well as on its excretion in milk. Contrasting data have been reported on the in vitro formation of AFL as an AFB1 metabolite in cattle liver. Using an HPLC technique, Kuilman et al. [45] detected AFM1 but not AFL in a bovine primary hepatocyte culture. Conversely, Pauletto et al. [24] were able to detect AFL formation by a more sensitive technique (LC-MS/MS) in a fetal bovine hepatocyte cell line (BFH12) even at a two-fold higher concentration with respect to AFM1. In AFB1-dosed goats, milk excretion of AFM1 largely prevailed, with AFL being present in trace amounts only [46]. Likewise, the administration of a single oral dose of AFB1 (300 mg) to lactating cows led to the milk excretion of both AFM1 and AFL with a concentration ratio of approximately 10:1 [47]. Considering a daily intake of 20 kg feed with an average AFB1 content of 5 μg/kg, cows from the present study were subjected to an estimated daily exposure of about 100 μg AFB1 for 10 days. Although under these conditions AFL excretion showed a spotted pattern, in the few cases in which the metabolite could be quantitated, we found AFL milk concentrations in the range 14–26 ng/L, lower than but of the same order of magnitude of those pertaining to AFM1 (range 36–66 ng/L). The different administration protocol (i.e., single vs. repeated doses) might explain the partial discrepancy with the results of the previous in vivo experiments mentioned above. 

Overall, the repeated exposure of dairy cows to AFB1 dietary levels at the upper EU regulatory limits was reflected by limited AFL milk excretion not affecting all exposed animals and reaching measurable concentrations (ng/L) in just a few of them. Such results are in sharp contrast with a survey on pasteurized retail dairy milk conducted in Mexico where 13% of the samples were found to contain AFL levels > LOQ (50 ng/L) and 8% with AFL levels >500 ng/L, with a peak of 12,400 ng/L [48]. 

It is worth noting that AFL has not yet been subjected to any risk assessment by EU or non-EU regulatory bodies, and it is not included in the routine chemical analyses for AF contamination of milk and milk products. Although AFL is generally considered less toxic than its precursor, it may act as a reservoir of AFB1, and in species like trout, the carcinogenic potency displayed by AFL is almost the same as that of AFB1 [49]. As regards the lack of measurable AFL levels in milk from TP-supplemented cows, further studies with a larger number of animals are needed to confirm this putative positive effect.

## 3. Conclusions

The increase in AFB1 maize contamination due to climatic change has prompted several research groups to look for natural phytogenic compounds as an alternative to mineral binders in the attempt to counteract the negative effects of the mycotoxin. Positive results from in vitro experiments pointed to the ability of TP (i.e., CUR and CURS) to inhibit the synthesis of AFM1 in AFB1-treated bovine cell lines.

Dairy cows offered a diet containing AFB1 concentrations at the EU maximum limits in compound feed for 10 days showed AFM1 milk levels approaching or exceeding the EU statutory limits and some spotted presence of AFL. Milk characteristics and milk yield were not affected by the treatment. The dietary supplementation of TP at a concentration within the levels considered safe by EFSA, however, was not able to significantly lower the AFM1 milk content. Since bioavailability could be an issue, further studies are warranted with a more bioavailable TP formulation.

## 4. Materials and Methods

### 4.1. Experimental Design and Sample Collection

The experimental design was evaluated by the Animal Welfare Body of the University of Bologna and found as not falling within the Directive 63/2010 of the European Parliament and of the Council on the protection of animals used for scientific purposes (transposed into Italian law by Legislative Decree 26/2014), thus not requiring any authorization from the national competent Authorities. The study was approved by the Animal Ethics Committee of the University of Bologna and conducted at the dairy cattle farm of the same university. Animals were 8 lactating Holstein–Friesian cows (average weight 752 ± 52 kg) in their late lactation period (262 ± 49 days in milk), with an average milk yield of 19.6 ± 6.6 kg/day. Cows were housed in a naturally ventilated free stall barn and had free access to feed and water. Rations were formulated to mimic total mixed rations used in the Parmigiano Reggiano cheese production area, in Italy, which consisted of all dry and nonfermented components, as detailed in previous studies [50,51]. 

Animals were divided into two groups, consisting of 4 cows each, and two different treatments of 10 days’ duration were performed. Group AFB1 received, from day 0 to day 2 only, the basal diet (AFB1 content less than 2 ng/g, see Section 4.3), while group TP was offered the basal diet supplemented with 20 g turmeric powder dissolved in linseed oil/head/day for 10 consecutive days. At day 3, all cows received the basal diet containing naturally contaminated maize with a final AFB1 concentration of 5 ± 1 μg/kg for 8 consecutive days. A crossover experimental design was applied: each cow received both treatments sequentially after of a 4-day washout period, during which all animals were offered the basal diet. The used TP contained 2.5% curcumin, desmethoxycurcumin and bis-desmethoxycurcumin (85/10/5). With an average feed intake of 20 kg/cow/day, the cows approximately received 0.5 g active substances/head/day; this dosage is higher than the recommended inclusion level for flavoring purposes but below the maximum safe concentration of 0.72 g/day calculated by the EFSA [7]. 

**Figure 2:**
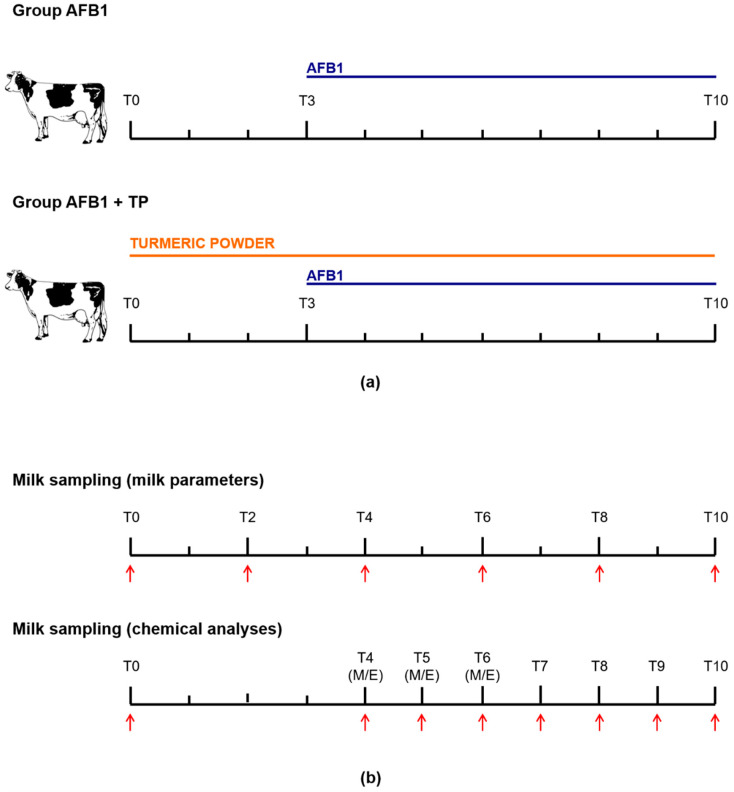
(**a**) Experimental design and (**b**) milk sampling time-table. M = morning; E = evening.

Animals were milked twice a day, namely at 08:00 h (referred to as M) and 19:30 h (referred to as E) in a double-5 herringbone milking parlour. Individual milk samples (around 100 mL) collected at T0, T2, T4, T6, T8 and T10 were used for the determination of somatic cell count (SCC) (Fossomatic 7, Foss Electric A/S, Hillerød, Denmark) and milk composition (fat, lactose, protein, urea) by means of infrared spectroscopy (MilkoScan FT+, Foss Electric A/S, Hillerød, Denmark). The determination of AFB1 metabolites was performed on further milk samples (around 100 mL) collected at T0 (M), T4, T5 and T6 (M and E), and T7, T8, T9 and T10 (M only), which were stored frozen (−20 °C) pending analysis. The experimental design is outlined in Figure 2.

### 4.2. Chemicals

High purity analytical standards of AFM_1_ and AFL were purchased from Toronto Research Chemicals (Toronto, ON, Canada), while ^13^C_17_-AFM_1_ was purchased from OrSell (Modena, Italy). All solvents employed for analysis were of LC-MS grade: acetonitrile, ammonium formate and formic acid were purchased from Sigma Aldrich (Milano, Italy), while ultrapure water was produced in-house each day of analysis (Millipore, Milano, Italy). Turmeric powder was purchased by BIORAMA (Rogeno, Italy).

### 4.3. Analytical Determinations

To measure AFM1 and AFL content in milk, an extraction on immunoaffinity columns (IAC) was performed, followed by analysis in ultra-performance liquid chromatography coupled with tandem mass spectrometry (UPLC-MS/MS). From each milk sample, a 100 mL aliquot was agitated on a vortex shaker for 15–20 s and transferred to two 50 mL polypropylene tubes, which were then centrifuged at 3000× *g* for 15 min at 4 °C. The obtained skimmed milk was filtered on a Whatman GF/A 150 mm filter (Fisher Scientific Italia, Milan, Italy), and 20 μL of the internal standard solution (^13^C_17_-AFM1 50 ng/mL in acetonitrile) was added to 50 mL of filtrate. The sample was placed into a 50 mL polypropylene syringe attached to a previously equilibrated (at least 1 h at room temperature) AflaClean M1 Select IAC column (LCTech, Obertaufkirchen, Germany) and left flowing by gravity or helped by applying a slight vacuum in case of excessive slowdown due to obstruction. To wash the column, 3 mL of water was loaded three times, and high vacuum was applied for 5 min. The analytes were recovered with 3 mL of methanol and centrifuged for 10 min at 1500× *g*, then 2.5 mL of the supernatant was evaporated to dryness with a UniVapo vacuum concentrator (UniEquip, Planegg, Germany). The dried residue was finally reconstituted in 200 μL of a water:methanol 90:10 (*v/v*) solution containing 5 mM ammonium formate and 0.1% formic acid, transferred into a vial for chromatography and analyzed.

The UPLC-MS/MS system consisted of a Waters Acquity UPLC binary pump coupled to a Waters Quattro Premier XE triple quadrupole mass spectrometer (Waters, Milford, MA, USA). Chromatographic separation was achieved with a Waters Acquity BEH C18 column (50 × 2.1 mm, 1.7 μM), kept at 40 °C. Mobile phase consisted of a variable mixture of water and methanol with 5 mM ammonium formate and 0.1% formic acid, flowing at 0.4 mL/min under programmed conditions during a 5 min chromatographic run. The spectrometer operated in positive electrospray mode (ESI+) with a capillary voltage of 3.75 kV and source and desolvation gas temperatures of 120 and 350 °C, respectively. The quantification and confirmation transitions (and related cone voltage and collision energy values) monitored for each analyte were: 329.1 > 272.8 *m*/*z* (42 V; 26 eV) and 329.1 > 228.9 *m/z* (42 V; 45 eV) for AFM_1_, 331.1 > 272.9 *m*/*z* (40 V; 25 eV) and 331.1 > 258.9 *m*/*z* (40 V; 26 eV) for AFM_2_, and 297.2>268.5 *m/z* (48 V; 20 eV) and 297.2 > 114.9 *m*/*z* (48 V; 63 eV) for AFL. For the internal standard ^13^C_17_-AFM_1_, the 346.2 > 288.4 *m/z* (44 V; 25 eV) transition was monitored. The limit of quantification was 12 ng/mL for AFL and 5 ng/L for AFM_1_. A matrix-matched calibration curve and quality control (QC) samples were freshly prepared for each day of analysis to assess method performances. The linearity was proved by the correlation coefficient (R2), always >0.99, and all the calibration standards within ±15% of the nominal value for all target analytes. Intraday accuracy and precision were in the range 4.1–6.7% and 8.4–9.6% for AFM_1_, 3.7–7.5% and 7.2–9.0% for AFM_2_, and 5.4–9.4% and 8.8–10.6% for AFL.

The AFB1 concentration in feed was determined by an external laboratory using an accredited LC-MS/MS method [52] with a LOQ of 2 ng/g.

### 4.4. Statistics

Data are expressed as mean ± SD or SEM. Normal distribution of data was assessed according to the D’Agostino and Pearson normality omnibus test. Significant differences were evaluated by two-way analysis of variance (ANOVA) for repeated measures, followed by Sidak’s multiple comparison test. Differences were considered statistically significant when the two-sided *p* value was <0.05. Analyses were performed with the GraphPad Prism 7.03 software (Graph Pad Software, San Diego, CA, USA).

## Figures and Tables

**Table 1:** Milk parameters from dairy cows of groups AFB1 and AFB1 + TP at T0, T4 and T10.

Parameter	T0		T4		T10	
	AFB1	AFB1 + TP	AFB1	AFB1 + TP	AFB1	AFB1 + TP
Milk yield (L)	19.1 ± 4.5	19.7 ± 4.8	17.7 ± 4.6	20.16 ± 4.7	16.9 ± 3.9	17.8 ± 4.1
Lipids (g/100 g)	3.37 ± 0.76	3.64 ± 0.52	3.46 ± 0.61	4.15 ± 1.52	4.08 ± 0.76	3.52 ± 0.75
Proteins (g/100 g)	3.44 ± 0.32	3.55 ± 0.37	3.52 ± 0.39	3.48 ± 0.34	3.58 ± 0.39	3.53 ± 0.38
Lactose (g/100 g)	4.54 ± 0.60	4.70 ± 0.23	4.55 ± 0.56	4.57 ± 0.16	4.57 ± 0.29	4.78 ± 0.42
Somatic cells (cell/mL × 1000)	258 ± 160	203 ± 97	187 ± 106	316 ± 246	323 ± 211	193 ± 117
Urea (mg/dL)	23.4 ± 6.8	24.4 ± 5.6	25.0 ± 6.2	27.1 ± 7.9	25.9 ± 4.0	23.6 ± 4.8

Data are expressed as mean ± SD (*n* = 8).

**Table 2:** AFL concentration in milk (ng/L) from cows administered AFB1 or AFB1 + TP.

	Time Points	Cow 1	Cow 2	Cow 3	Cow 4	Cow 5	Cow 6	Cow 7	Cow 8
AFB1 + TP	T4M	n.d.	n.d.	n.d.	n.d.	n.d.	n.d.	n.d.	n.d.
	T4E	n.d.	n.d.	<LOQ	n.d.	n.d.	n.d.	n.d.	n.d.
	T5M	n.d.	n.d.	<LOQ	n.d.	n.d.	n.d.	<LOQ	n.d.
	T5E	n.d.	n.d.	n.d.	n.d.	n.d.	n.d.	n.d.	n.d.
	T6M	<LOQ	n.d.	n.d.	n.d.	n.d.	n.d.	<LOQ	n.d.
	T6E	<LOQ	n.d.	n.d.	n.d.	n.d.	n.d.	n.d.	n.d.
	T7M	n.d.	n.d.	n.d.	n.d.	n.d.	n.d.	n.d.	n.d.
	T8M	n.d.	n.d.	n.d.	n.d.	n.d.	n.d.	<LOQ	n.d.
	T9M	n.d.	n.d.	n.d.	n.d.	n.d.	n.d.	n.d.	n.d.
	T10M	n.d.	n.d.	n.d.	n.d.	n.d.	n.d.	n.d.	n.d.
AFB1	T4M	n.d.	n.d.	n.d.	n.d.	n.d.	n.d.	n.d.	<LOQ
	T4E	n.d.	n.d.	n.d.	n.d.	n.d.	n.d.	n.d.	<LOQ
	T5M	n.d.	n.d.	n.d.	n.d.	n.d.	n.d.	n.d.	n.d.
	T5E	n.d.	<LOQ	n.d.	n.d.	n.d.	n.d.	19	n.d.
	T6M	n.d.	n.d.	n.d.	n.d.	n.d.	n.d.	n.d.	n.d.
	T6E	n.d.	n.d.	<LOQ	n.d.	n.d.	n.d.	n.d.	n.d.
	T7M	<LOQ	14	n.d.	n.d.	n.d.	n.d.	n.d.	n.d.
	T8M	n.d.	n.d.	n.d.	n.d.	n.d.	n.d.	26	n.d.
	T9M	n.d.	n.d.	n.d.	n.d.	n.d.	n.d.	21	n.d.
	T10M	n.d.	n.d.	n.d.	n.d.	n.d.	n.d.	n.d.	n.d.

LOQ: Limit of Quantification (12 ng/L); n.d. = not detectable (under the Limit of Detection, LOD).

## Data Availability

All the data analyzed during this study are included in this published article and in its Supplementary Materials.

## Supplementary Materials

The following supporting information can be downloaded at: https://www.mdpi.com/article/10.3390/toxins14070430/s1, Table S1: Milk parameters from dairy cows of groups AFB1 and AFB1 + TP at T2, T6 and T8.
