# Supplementary material for: Effects of Turmeric Powder on Aflatoxin M1 and Aflatoxicol Excretion in Milk from Dairy Cows Exposed to Aflatoxin B1 at the EU Maximum Tolerable Levels
Source: Toxins (Basel). 2022 Jun 24;14(7):430. doi: 10.3390/toxins14070430 (PMC9317782; doi:10.3390/toxins14070430)
Supplement: Supplementary file 1 [file toxins-14-00430-s001.zip › toxins-1773959-SI.pdf]

toxins

MDPI

Effects of Turmeric Powder on Aflatoxin M1 and Aflatoxicol Excretion in Milk from Dairy Cows Exposed to Aflatoxin B1 at the EU Maximum Tolerable Levels

Flavia Girolami, Andrea Barbarossa, Paola Badino, Shiva Ghadiri, Damiano Cavallini, Anna Zaghini and Carlo Nebbia

Table S1. Milk parameters from dairy cows of groups AFB1 and AFB1 + TP at T2, T6 and T8.

Parameter	T2		T6		T8	
	**AFB1**	**AFB1 + TP**	**AFB1**	**AFB1 + TP**	**AFB1**	**AFB1 + TP**
Milk yield (L)	18.2 ± 4.4	18.8 ± 3.7	17.5 ± 5.5	18.5 ± 5.6	17.6 ± 4.0	18.2 ± 5.3
Lipids (g/100g)	3.70 ± 0.52	3.71 ± 0.52	3.80 ± 0.61	3.71 ± 0.52	3.70 ± 0.67	4.29 ± 1.12
Proteins (g/100g)	3.47 ± 0.29	3.5 ± 0.32	3.51 ± 0.29	3.51 ± 0.33	3.53 ± 0.32	3.56 ± 0.36
Lactose (g/100g)	4.62 ± 0.26	4.65 ± 0.29	4.51 ± 0.54	4.64 ± 0.19	4.64 ± 0.24	4.61 ± 0.21
Somatic cells (cell/mL*1000)	276 ± 121	222 ± 156	243 ± 145	286 ± 196	244 ± 145	254 ± 117
Urea (mg/dL)	27.5 ± 7.6	27.0 ± 4.9	26.4 ± 7.0	25.6 ± 5.1	28.6 ± 7.1	29.3 ± 5.1

Data are expressed as mean ± SD.
